# The gut microbiome in human immunodeficiency virus infection

**DOI:** 10.1186/s12916-016-0625-3

**Published:** 2016-06-03

**Authors:** Gili Zilberman-Schapira, Niv Zmora, Shlomik Itav, Stavros Bashiardes, Hila Elinav, Eran Elinav

**Affiliations:** Department of Immunology, Weizmann Institute of Science, 234 Herzl Street, Rehovot, 76100 Israel; Hadassah AIDS Center, Department of Clinical Microbiology and Infectious Diseases, Hadassah-Hebrew University Medical Center, Jerusalem, 91120 Israel

**Keywords:** Microbiota, Dysbiosis, Gastrointestinal tract, AIDS, HIV, Anti-retroviral therapy, CD4+ T cells

## Abstract

HIV/AIDS causes severe dysfunction of the immune system through CD4+ T cell depletion, leading to dysregulation of both the adaptive and innate immune arms. A primary target for viral infection is the gastrointestinal tract, which is a reservoir of CD4+ T cells. In addition to being a major immune hub, the human gastrointestinal tract harbors trillions of commensal microorganisms, the microbiota, which have recently been shown to play critical roles in health. Alterations in the composition and function of microbiota have been implicated in a variety of ‘multi-factorial’ disorders, including infectious, autoimmune, metabolic, and neoplastic disorders. It is widely accepted that, in addition to its direct role in altering the gastrointestinal CD4+ T cell compartment, HIV infection is characterized by gut microbiota compositional and functional changes. Herein, we review such alterations and discuss their potential local and systemic effects on the HIV-positive host, as well as potential roles of novel microbiota-targeting treatments in modulating HIV progression and associated adverse systemic manifestations.

## Background

Since the emergence of the acquired immunodeficiency syndrome (AIDS) epidemic in the early 1980s and the discovery of its causative agent, the human immunodeficiency virus (HIV), the deleterious effects of HIV infection on the human immune system, and mainly on the CD4+ T cell compartment, have been extensively studied. Upon the advent of highly active antiretroviral therapy (HAART), the burden of HIV-related morbidity and mortality dropped dramatically, although complete elimination of the disease has not yet been achieved. Additionally, effective antiretroviral therapy (ART) is accompanied by the emergence of long-term HIV- and treatment-related metabolic manifestations, suggesting that significant gaps remain in our comprehension of the mechanisms by which the virus exerts adverse effects on its host. Considering that 60 % of CD4+ T cells in the human body are estimated to reside in gut-associated lymphoid tissue, and since the reconstitution of these cell populations and the gut microbial composition is incomplete even under HAART, the human intestine has recently become the focus of attention in HIV research. Moreover, changes in gut microbial composition and function in HIV-positive individuals, aside from being secondary to HIV infection, may also play direct roles in mediating some disease manifestations. Potential mechanisms for such effects include local facilitation of viral infection, creation of viral sanctuary sites resistant to systemic ART treatment, and promotion of impaired gut mucosal barrier function, resulting in leakage of bacterial components into the main circulation. These mechanisms may potentially contribute to the long-term immune, infectious, metabolic, and neoplastic manifestations of HIV infection, collectively termed the ‘inflamm-aging’ of HIV. Herein, we focus on the intricate interactions between HIV, host gastrointestinal tract, and gut microbiota, and highlight how these interactions may contribute to immune and non-immune consequences on the host.

### Immunological aspects of intestinal HIV infection

HIV infection is characterized by three, partially overlapping clinical stages – an acute phase, a chronic latency phase and a late advanced stage – during which clinical manifestations related to host immune deficiency emerge [1]. CD4+ T cell depletion characterizes all stages of the disease. The driving force behind the gradual CD4+ T cell depletion is chronic systemic immune activation, which increases co-receptor expression and allows viral cell entry into previously uninfected T cells [2], thus leading to cell death through a viral cytopathic effect and cytotoxic T cell attack of infected cells [3]. Latent HIV infection involves features of immune activation and dysregulation, which may be useful in complementing direct viral load measurements in assessing a patient's clinical status [4]. Late HIV stage is characterized by an increase in viral load and a decrease in circulating CD4+ T cell levels, typically to below 200 cell/mm^3^, as well as by the emergence of multiple opportunistic infections and immune deficiency-related neoplastic and neuro-degenerative disorders [5]. Over the past two decades, combined ART treatment has succeeded in reducing plasma viral loads to undetectable concentrations whilst restoring CD4+ T cell levels in peripheral blood, thus dramatically reducing – though not eliminating – the prevalence of an AIDS-related pathology in treated patients. A detailed description of the clinical course of HIV and AIDS is concisely described elsewhere [6].

Studies focusing on CD4+ T cell depletion and its direct contribution to HIV-related immune deficiency mostly assess easily accessible peripheral blood samples [7–9]. However, the initial and most pronounced depletion of CD4+ T cells commonly occurs in the gastrointestinal mucosa during the acute phase of HIV infection [10], partially due to the high concentration of CCR5 co-receptor-expressing CD4+ T cells within the gut, allowing HIV entry and replication [9]. CD4+ T cell depletion in the acute phase of HIV infection is less pronounced in peripheral blood compared to the gastrointestinal tract, possibly stemming from the presence of CCR5-low CD4+ T cell populations within the peripheral blood compartment [9]. Infective simian immunodeficiency virus models have indicated that this rapid intestinal CD4+ T cell depletion occurs within as little as 7 days post-infection [11].

Chronic HIV infection within the gastrointestinal tract, as well as the closely associated secondary reduction in CD4+ T cells, significantly affect gut physiology. Whilst allowing the digestion and absorption of luminal nutrients into the host, epithelial cells in the mucosal epithelia of the gastrointestinal tract simultaneously prevent the activation of a systemic immune response triggered by commensal microorganisms [12]. HIV infection leads to a dysregulation of the gastrointestinal immune-epithelial network [12]; early in acute HIV infection, mucosal infiltration with activated memory CD4+ and CD8+ T cells leads to apoptosis and impaired barrier function [13], mediated by tight junction disruption through the secretion and activation of pro-inflammatory cytokines such as TNF-α, IL-6, and IL-8 [14]. Further, gut HIV infection causes depletion and dysfunction of key resident immune populations, such as Th17 and CD103+ dendritic cells, in addition to CD4+ T cell depletion [12]. HIV infection may also lead to lower levels of IgA, possibly contributing to HIV-associated enhanced microbial translocation, which may lead, in turn, to a chronic state of immune activation as noted in many HIV carriers [15]. Enhanced gut microbial translocation is exemplified by an elevation in plasma systemic lipopolysaccharide (LPS) levels in HIV-positive patients [16] Elevated LPS levels have also been correlated with activated CD8+ T cell numbers [16] and have been suggested to drive chronic stimulation of monocytes.

During progression to AIDS, further immune dysfunction, at both the local gut and systemic levels, results in a marked propensity to develop infectious diarrhea in up to 90 % of patients [17]. AIDS-associated gastrointestinal symptoms are driven by both ‘regular’ pathogens, such as *Escherichia coli*, *Salmonella*, and *Shigella*, and opportunistic ‘pathobionts’, such as *Cryptosporidium*, *Cytoisospora belli*, *Microsporidium*, *Cytomegalovirus*, and *Micobacterium avium-intracellulare* [18, 19], all of which lead to severe morbidity and mortality [20]. In addition, treatment for HIV infection or its complications is often associated with a variety of gastrointestinal symptoms [21]. Thus, HIV infection has a profound effect on gut immune function as characterized by CD4+ T cell depletion and immune modulation.

### HIV and the gut microbiota

The human gastrointestinal tract harbors a complex microbial ecosystem within its epithelial cell lining, with microorganisms outnumbering host cells by a factor of 10–100 [22]. As a normal gut microbiota is essential for immune homeostasis; disruptions in intestinal immunity can precipitate gut dysbiosis, which may in turn induce chronic inflammation in the mucosa and periphery. The latter (summarized in Table 1) is commonly observed in HIV-positive individuals [23, 24].

**Table 1:** Summarizing Microbiota in HIV Table

Year	Technique	Study Groups	Main findings
Ellis et al. 2011 [33]	Kinetic (real time) quantitative-PCR of the 16S rRNA gene	12 HIV positive individuals, 5 seronegative individuals.	• No significant difference in the total quantity of 16S rRNA gene expression between HIV positive and seronegative individuals. • A greater proportion of gram-negative bacteria, order Enterobacteriales was seen in HIV positive individuals compared with seronegative controls. • The proportions of Enterobacteriales and Bacteroidales correlated with duodenal CD4+ T-cell depletion and peripheral CD8+ T-cell activation, respectively.
Vujkovic-Cvijin et al. 2013 [32]	Microarray of 16S rRNA gene	22 HIV positive individuals (6 viremic untreated, 16 on HAART), 9 seronegative individuals	• Dysbiotic mucosal-adherent microbiota. Enrichment of Proteobacteria and depletion of Bacteroidia in HIV infected individuals. • Dysbiosis in individuals on HAART was correlated with the kynurenine pathway of tryptophan catabolism and plasma concentrations of IL-6
Lozupone et al. 2013 [27]	V4 region of 16S rRNA gene	22 chronic HIV infected individuals (with or without ART), 3 recently infected HIV positive individuals, 13 HIV seronegative individuals	• Recently infected individuals have a microbiota that differs only slightly from the microbiota of uninfected individuals. • Short-term ART did not restore the microbiota to its uninfected composition. • Increase abundances of *Prevotellaceae*, *Erysipelotrichaceae*, *Veillonellaceae*, *Clostridium* cluster XIII and the genus *Desulfovibrio* in chronically untreated individuals compared to HIV seronegative individuals. • HIV seronegative individuals had increased abundance of *Bacteroidaceae*, *Rikenellaceae*, and *Porphyromonadaceae*.
McHardy et al. 2013 [39]	V4 region of 16S rRNA gene, in-silico metagenomics	20 HIV seronegative individuals, 20 HIV positive individuals on ART, 20 HIV positive individuals not on ART.	• Depletion of specific genera (such as *Lachnospira,* and *Eubacterium,*) and enrichment of other genera (such as *Porphyromonas* and *Anaerococcus*) in HIV positive individuals not on ART. • HIV positive individuals on ART showed similar trends but to a lesser extent. • Differences in attributed functionality were found between HIV positive individuals not receiving ART and healthy controls (eg. amino acid metabolism and vitamin biosynthesis).
Pérez-Santiago et al. 2013 [35]	V6 region of 16S rRNA gene	13 HIV positive individuals (before and during ART)	• Enrichment of Lactobacillales in HIV infected individuals before ART was associated with lower viral loads, higher CD4 T cell concentrations and lower markers of microbial translocation. • Enrichment of Lactobacillales in HIV infected individuals after ART initiation was associated with lower translocation, lower systemic immune activation and higher CD4 T cell concentrations
Mutlu et al. 2014 [29]	16S rRNA gene	21 HIV positive individuals, 22 seronegative individuals.	• Reduced alpha diversity in the terminal ileum and colon were observed in HIV infection. • Bacteria that increased in the HIV positive group are potentially pathogenic in other disease states. • Higher diversity between microbiota samples in the HIV positive group compared with the seronegative group. • A significant increase in *Brachyspira*, *Campylobacter*, *Catenibacterium*, *Escherichia*, *Mogibacterium*, *Prevotella*, and *Ralstonia* was observed in the HIV positive group. • An increase in *Akkermansia*, *Bacteroides*, *Blautia*, *Coprococcus*, *Dialister*, *Dorea*, *Faecalibacterium*, *Lachnospira*, *Roseburia*, *Ruminococcus*, *Odoribacter*, *Oscillospira* was observed in the seronegative individuals group.
Lozupone et al. 2014 [24]	V4 region of 16S rRNA gene	40 HIV positive individuals (of them 28 on ART), 15 HIV seronegative individuals.	• The microbiota composition of individuals on ART was more similar to that of individuals with untreated HIV infection than seronegative individuals. • *Bacteroides* and *Odoribacter* genera and *Parabacteroides distasonis* decrease with HIV infection, remain at low abundances in most individuals on ART. • *Prevotella* genus, the *Paraprevotellaceae* family, and *Eubacterium biforme* increase with HIV infection, abundance varies in individuals undergoing ART (do not reach typical low levels of HIV-negative individuals). • *Peptococcus* genus increased in untreated HIV infected individuals and decreases with ART. • *Desulfovibrio* and *Catenibacterium* genera increase in untreated HIV infected individuals, while in individuals on ART they trended back to levels seen in HIV seronegative individuals.
Dillon et al. 2014 [28]	V4 region of 16S rRNA gene	18 untreated HIV positive individuals, 14 seronegative individuals	• Increased abundance of Proteobacteria and decreased abundance of Firmicutes in colon biopsies of HIV infected individuals compared with seronegative individuals. • Within the Proteobacteria phylum, an increase in *Brucellaceae*, *Xanthomonadaceae* and *Moraxellaceae* and a decrease in *Rhodospirillaceae* was observed in HIV infected individuals. • Within the Bacteroidetes phylum, an increase in Prevotellaceae and a decrease in *Bacteroidaceae*, *Prophyromonadaceae* and *Rikenellaceae* was observed HIV positive individuals. • Within the Firmicutes phylum, *Lachnospiraceae*, *Christensenellaceae* and *Ruminococcaceae* were decreased in HIV-infected patients. • The increased abundance in Proteobacteria seen in mucosal samples of HIV positive individuals, was not observed in fecal aspirates or rectal swabs. • The decrease in mucosal Firmicutes in HIV infected individuals was observed in fecal aspirates, but not in stool samples. • Trends seen in mucosal abundances of Proteobacteria and Firmicutes families and genera were not consistent in stool samples and fecal aspirates.
Nowak et al. 2015 [26]	V3-V4 region of 16S rRNA gene	31 HIV positive individuals (28 viremic, 3 elite controllers), 9 HIV seronegative individuals	• Decreased alpha diversity in untreated HIV infected patients. • A further decrease in alpha diversity following ART. • Prevotella genus significantly reduced during ART in HIV positive individuals. • Higher relative abundance of Bacteroidetes in elite controllers compared to viremic patients. • Increased abundance of Actinobacteria and Proteobacteria in viremic patients compared to elite controllers. • Elite controllers did not differ significantly from seronegative controls at the phylum level. • Increased relative abundance of *Lactobacillus* in viremic patients compared to seronegative individuals. • *Lachnobacterium*, *Faecalibacterium*, and *Haemophilus* were significantly reduced in viremic patients compared to seronegative individuals.
Vázquez-Castellanos et al. 2015 [37]	V1, V2, V3 regions of 16S rRNA gene, whole genome shotgun sequencing	15 HIV positive individuals on ART, 15 seronegative individuals	• Healthy subjects cluster separately from positive subjects based on 16S rRNA sequencing. • Enrichment in *Prevotella* and *Succinivibrio* in the microbiota of HIV positive individuals. • Seronegative control individuals presented with microbiota enriched with *Bacteroides* and *Faecalibacterium* and generally had a more diverse composition. • 12 KEGG pathways found to be enriched in the HIV positive subjects, specifically “ribosome” and “LPS biosynthesis” pathways being the most discriminative. • 23 KEGG pathways found to be enriched in seronegative control, “starch and sucrose metabolism” and “glycolysis/gluconeogenesis” being the most discriminative.
Dinh et al. 2015 [23]	V3-V5 region of 16S rRNA gene	21 HIV positive individuals on ART, 16 seronegative individuals	• Greater abundance of Proteobacteria in HIV positive individuals compared to controls. • Enrichment in Gammaproteobacteria, Enterobacteriales and *Enterobacteriaceae* in the Proteobacteria phylum in HIV positive individuals compared to controls. • Enrichement in Erysipelotrichi, Erysipelotrichales, and *Erysipelotrichaceae* in the Firmicutes phylum in HIV positive individuals compared to controls. • Enrichement in *Barnesiella* and reduction in *Rikenellaceae* and *Alistipes* in the phylum Bacteroidetes in HIV positive individuals compared to controls.
Monaco et al. 2016 [36]	V4 region of 16S rRNA gene	40 HIV positive individuals on ART, 42 HIV positive individuals not on ART, 40 HIV seronegative individuals	• Low peripheral CD4 T cell counts associated with reduced phylogenetic diversity and species richness. • Low peripheral CD4 T cell concentration associated with an increase in a group of bacteria such as *Enterbacteriaceae*, *Enterococcaceae* and *Lactobacillaceae*.

Sequencing of the highly conserved 16S rDNA gene, which features species-specific highly variable regions, enables the characterization of genus-, and at times, species-level relative microbiota compositions [25]. Emerging evidence from 16S rDNA sequencing of stool samples of HIV-negative, HIV-positive, and ART-treated infected individuals indicated that the gut microbiota differs between these populations [26, 27]. In the ART-treated population, while viral suppression was noted, the microbiota composition was not completely restored to its HIV negative state. Nevertheless, the exact microbiome configuration in HIV-positive individuals, ART-treated patients and healthy controls remain inconsistent between studies.

**Fig. 1:**
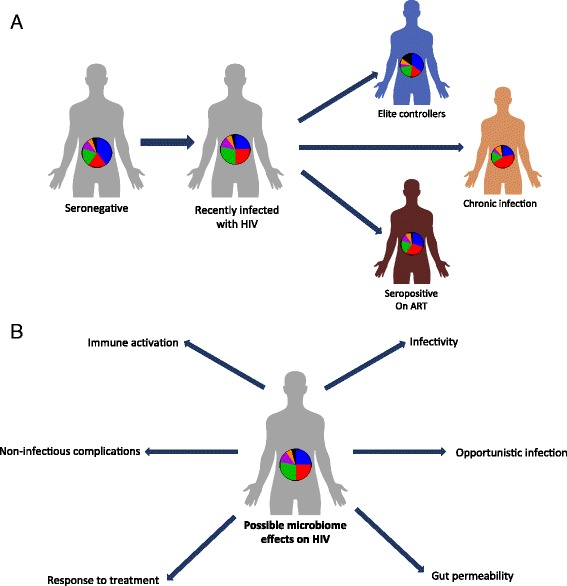
Gut microbiota alterations during HIV infection and their potential effects on the host. **a**. In different studies, distinct gut microbiome compositions have been identified in HIV infected individuals with or without ART, as compared to healthy controls. Importantly, HIV-associated microbiome configurations vary between these studies. While ART dramatically lowers the viral load in infected individuals, gut microbiome composition is not fully restored to a healthy composition. ‘Elite controllers’ differ in their microbial composition from HIV- infected individuals and are more similar to healthy individuals. **b**. The characteristic HIV microbiota possibly contributes to some of the common HIV manifestations, including modification of the level if infectivity, occurrence of opportunistic infections, increased gut permeability and resultant bacteria and bacterial product translocation, increased immune activation and T cell polarization, metabolic complications and variability in the response to HIV treatment

When considering bacterial composition as a whole, rather than focusing on specific taxa, a variety of studies identified different patterns of composition for each of the study groups (summarized in Fig. 1a). Lozupone et al. [27] demonstrated that HIV-negative and chronically infected HIV-positive individuals have distinguishable gut microbial compositions. One recurring finding is that the *Prevotella* genus is significantly enriched in HIV-positive gut microbiota compared with HIV-negative individuals; however, to date, the effects of this microbial expansion remain to be elucidated [27–29]. Nowak et al. [26] suggest that the distinct microbial compositions within HIV-positive individuals may stem from differences in HIV viral load. In a small cohort, they demonstrate that elite controllers, i.e., HIV-positive individuals with controlled viral load, have a microbial composition that is distinct from that of viremic patients, and overall more similar to healthy controls. Of note, some of the above studies utilized stool samples, which are known to be representative of the lumen microbiota composition. The mucosal microbiota may be more clinically relevant due to its close proximity to the host intestinal niche. Nevertheless, mucosal microbiota differs greatly from the luminal fraction, even within the same individual [30, 31]. It is therefore unsurprising that different patterns emerge when considering the mucosal microbiota of HIV-positive and HIV-negative individuals. Vujkovic-Cvijin et al. [32] investigated the changes in mucosal microbial composition in recto-sigmoid biopsy specimens from HIV-negative, HIV-positive, and ART-treated subjects using a bacterial 16S rRNA probe microarray; the microbiota composition of the mucosal-adherent bacteria was found to differ between HIV-positive and HIV-negative individuals. Exploring other regions of the gut, Mutlu et al. [29] found that the different compositional patterns between HIV-positive and HIV-negative individuals were also present in samples obtained from the ileum and colon. Specifically, the HIV-positive terminal ileum and the colon featured reduced species richness (i.e., alpha diversity), while the luminal microbiota featured less pronounced differences. Dillon et al. [28] also investigated alterations in the colonic mucosal microbiota between HIV-positive individuals and healthy controls. Within the ten most abundant bacterial families, they found a significant increase in the Prevotellaceae and Ruminococcaceae families and a significant decrease in the Becteroidaceae and Lachnospiraceae families in HIV-positive individuals versus healthy controls. Ellis *et al.* [33] applied a slightly different approach of quantitative PCR of the 16S rRNA region. In this study the total quantity of universal 16S rRNA did not differ between the HIV positive and the control groups, however, a trend for a higher proportion of the gram-negative bacteria order Enterobacteriales in the HIV-positive group compared to control was demonstrated.

A more mechanistic understanding of these compositional changes has been provided by recent studies focusing on the association between disease progression and disease markers with specific bacterial taxa [27, 28]. Nevertheless, to date, CD4+ T cell count remains the primary laboratory marker used in the follow-up of disease progression in HIV [34]. The bacterial order Lactobacillales was found to be positively correlated with CD4+ T cell count, a correlation that persisted even after initiation of ART [35], while the abundance of the *Bacteroides* genus trended towards a negative correlation with CD4+ T cell count [28]. Another study indicated that higher CD4+ T cell counts positively correlate with higher bacterial diversity within a sample [26]. Recently, Monaco et al. [36] reported an association between immunodeficiency in HIV-positive individuals and alterations in the gut bacterial and viral component of the microbiome [36]. Low peripheral T cell counts were associated with an overall reduced phylogenetic diversity and richness in the gut bacterial populations, while there was an increase in abundance of specific bacteria such as Enterobacteriaceae, which have been associated with inflammation and may be a contributing factor in AIDS-related enteropathy [36]. Of note, some studies did not identify any correlation between specific taxa and CD4+ T cell counts [29, 32]. Several bacterial genera were found in lower abundances in HIV-positive individuals and correlated positively with protective immune markers (e.g., low T cell activation) [28, 32]. In contrast, markers of disease progression (e.g., pro-inflammatory cytokine levels) were found to be correlated with bacteria genera enriched in HIV-positive individuals [32]. Other studies linked viral load, CD4+/CD8+ ratio, systemic biomarkers, cytokines, immune activation, bacterial translocation, and thymic function to gut bacterial abundance in different taxonomical levels [26, 28, 29, 35, 37].

Next generation sequencing-based characterization of the total microbial DNA content (whole genome shotgun sequencing) allows the reconstruction of genes, pathways, and modules of various microbiota configurations and enables the characterization of the functional basis of microbiota dysfunction noted in HIV-infected individuals. By applying this method, Vázquez-Castellanos et al. [37] began to unravel the metabolic pathway alterations taking place in the dysbiotic environment and their association with immune activation and inflammation as observed in HIV-infected patients. One interesting finding was the LPS biosynthesis pathway, which was found to be enriched in the HIV-positive group and to be a discriminative factor between the HIV-positive and HIV-negative groups [37]. Moreover, they demonstrated that an increased prevalence of LPS biosynthesis genes is associated with a decrease in strain diversity and an increase in the proportion of gram-negative bacterial presence. Interestingly, an increase in plasma LPS concentration is an established marker of HIV infection progression, and LPS is a known immune-stimulant, possibly contributing to systemic immune activation in infected patients [38]. McHardy *et al*. [39] addressed the issue of changes in intestinal mucosal bacterial composition occurring during HIV infection and subsequently following ART by combining 16S rRNA sequencing with in-sillico methods to infer the functionality of the bacterial populations. By applying PICRUSt [40] and HUMAnN [41] to the 16S rRNA sequencing data they were able to infer not only changes in composition, but also to reveal changes in metabolic functional potential in pathways involving, among others, amino acid metabolism and vitamin biosynthesis, suggesting alterations in nutrient availability between the study groups.

Further, in a principal coordinate analysis performed in Vázquez-Castellanos et al. [37], HIV-positive versus control individuals depicted clear and statistically significant differences, highlighting a typical and specific microbiota composition in the HIV-positive as compared to the non-infected control group; these differences stem mainly from enrichment in *Prevotella* and *Succinivibrio* in the HIV-positive group, and *Bacteroides* and *Faecalibacterium* in healthy controls. When performing a correlation analysis between these typical compositions (namely the first principal component) and immunological markers of disease progression, some statistically significant correlations were identified. Specifically, the first principal component positively correlated HIV microbiota changes with C-reactive protein (an inflammation marker) levels and with markers of T cell activation such as CD4 + HLA–DR + CD38+, CD4 + CD25+, CD8 + HLA–DR + CD38+, and CD8 + CD38+ T cell percentages. While causality was not demonstrated in these associations, they may potentially point towards involvement of microbiota dysbiosis in immune alteration in HIV [37]. Mechanistic determination of potential causality between microbiota commensal changes and these immune phenomena merit further study.

Collectively, these preliminary studies suggest that HIV-driven interference to the delicate balance of gut mucosal immunity may cause a disruption of gastrointestinal tolerance, which may in turn promote dysbiosis in HIV-positive individuals. Dysbiosis can result in activation of immune cells and promotion of bacterial translocation leading to systemic inflammation, a hallmark of chronic HIV infection (Fig. 1b).

### Mucosal dysbiosis as a risk factor for HIV acquisition

The mucosal barrier confers effective protection against sexual transmission of HIV; indeed, only 0.1 % of unprotected receptive vaginal intercourses and 1.4–1.7 % of unprotected receptive anal intercourses result in the acquisition of the virus [42]. However, the presence of mucosal inflammation and immune activation increases the risk for HIV transmission [42, 43]; it is believed that tissue-residing bacteria may affect human mucosal immune function, which may modulate this risk [44]. An example of the effects of the microbiota–immune interface on HIV infectivity resides in vaginal microbiota, where dysbiosis (termed bacterial vaginosis) is suggested to enhance HIV infectivity, as observed in the marked increase in susceptibility for HIV acquisition and transmission in bacterial vaginosis patients [44]. This enhanced infectivity is associated with elevated pro-inflammatory cytokine concentrations, especially IL-1β, and CCR5 + CD4+ cell activation and recruitment in the female genital tract mucosa [45–47]. In contrast, an increase in the relative abundance of Lactobacilli, an important member of the ‘healthy’ indigenous vaginal microbiota, induces an anti-inflammatory state contributing to the relative resistance to HIV infection [48] Indeed, a recent study by Anathar et al. [47] categorized vaginal microbiota profiles (cervicotypes) to four distinct bacterial community structures. *Lactobacillus crispatus*-rich vaginal microbiota were found to manifest the lowest inflammation, while highly diverse communities, namely *Prevotella*-containing and to a lesser extent *Gardnerella*-dominant microbiota, correlated with multiple pro-inflammatory mucosal cytokines even in the absence of overt sexually transmitted diseases. Notably, similar to what has been previously described for the gut, successful HAART with serum viral load suppression does not fully attenuate HIV-induced immune activation in the female genital tract [49]. Intriguingly, semen dysbiosis was found to be correlated with pro-inflammatory semen cytokines and elevated HIV viral load in the semen, which implies that male genital tract dysbiosis may also play a role in HIV sexual transmission [50]. These noted associations between male and female reproductive microbial changes in the mucosa and the risk for HIV acquisition remain to be further explored, and will constitute an exciting future avenue in HIV research. Potentially, understanding these microbial effects on HIV transmissibility may assist in developing novel microbiota-associated HIV prevention strategies.

### Non-immune HIV manifestations and possible links to dysbiosis

The emergence of HAART rendered HIV infection a chronic disease. Nevertheless, despite viral load suppression and immune reconstitution, infected individuals still encounter a reduced life expectancy and increased morbidity and mortality. The chronicity of HIV introduced a new challenge to its follow-up and treatment, as chronically infected patients feature a variety of non-AIDS disorders associated with long-term viral infection, ART, immune dysregulation, and, potentially, microbiota dysbiosis [51]. Chronic HIV infection is characterized by enhanced atherosclerosis, leading to a propensity to develop cardiovascular diseases, including myocardial infarction, cerebrovascular disease [52], and peripheral vascular disease [53]. It has been demonstrated that the intestinal microbiota of HIV-negative subjects featuring increased metabolism of choline into trimethylamine (TMA), which is further oxidized to trimethylamine-N-oxide, may carry atherogenic potential [54]. Interestingly, a recent study showed that HIV-positive patients exhibit worsened calcified coronary plaque features compared to non-infected individuals, as visualized by cardiac computed tomography angiography [55] and that these radiologic features were correlated to serum TMA and LPS levels, potentially linking microbiota to these systemic disease risks [55]. Similarly, another recent study showed that serum trimethylamine-N-oxide levels were associated with silent ischemia in HIV-positive individuals [56]. HIV-positive patients exhibit immune activation leading to atherosclerosis even after the initiation of HAART [57]. It has been found that common carotid intima media thickness measured by ultrasonography correlated with sCD14 and IL-6 levels in serum. Additionally, it was implied that altered tryptophan catabolism could account for the increased cardiovascular disease risk, as the kynurenine-to-tryptophan ratio was elevated in patients who exhibited enhanced atherosclerosis. Tryptophan catabolism was shown to be associated with cardiovascular disease risk in other settings and to be indicative of poor immune reconstitution and of all-cause mortality in HIV-positive patients [32, 58], regardless of treatment status. Since this pathway is activated in response to interferons and bacterial products, dysbiosis may play a pivotal role in its alteration [59]. Collectively, these findings suggest that altered intestinal microbiota profiles may potentially contribute to cardiovascular disease in HIV-positive patients. This dysbiosis could be ascribed to enrichment in *Prevotella* [60], which possess TMA-production capabilities known to be increased in HIV-positive individuals [27], or depletion of species, such as *Akkermansia*, found to be protective against metabolic disorders [29, 61]. Additionally, a Proteobacteria-enriched and Bacteroidia-depleted mucosal-adherent colonic bacterial profile was proposed to stimulate kynurenine pathways of tryptophan catabolism and was associated with chronic inflammation and disease progression in HIV-infected individuals [32]. Furthermore, microbial translocation-induced systemic endotoxemia was found to trigger obesity, insulin resistance, diabetes mellitus, hypertension, dyslipidemia, atherosclerosis, and endothelial dysfunction, which may account for the increased cardiovascular risk seen in HIV-positive individuals [53].

Lipodystrophy, a metabolic disorder visually characterized by impaired distribution of body fat, is commonly seen in HIV-positive individuals. Increases in *Prevotella* and decreases in *Bacteroides* species in these patients were suggested to contribute to metabolic diseases, especially when consuming a high-fat diet [24, 27]. Other non-immune pathologies associated with HIV infection and which may be related to dysbiosis include hepatic damage due to the translocation of microbial products passing through the portal circulation [62]; HIV-associated ‘wasting syndrome’, characterized by malabsorption, low weight gain, and steatorrhea, and suggested to result from an abnormal interplay between the intestinal microbiota, an impaired immune response, and a dysfunctional gut epithelium leading to decreased metabolic activity [63]; and oral candidiasis, a common manifestation in advanced stages of HIV, resulting from dysregulation in quorum-sensing between oral bacterial and fungal microbiota [64]. Additionally, it has been suggested that the gut microbiota may modulate HIV latency and promote progression of AIDS by regulating the epigenetic status of integrated proviral DNA, an effect that could be mediated by butyric acid-producing bacteria [65]. All of these HIV manifestations, potentially modulated by microbiota changes, merit further mechanistic studies. As microbiota research progresses, the impact of gut bacteria on other extra-intestinal phenomena may be also unraveled, and missing links in the interplay between the host immune system and the virus may be found to be orchestrated or mediated by bacterial functions.

### Probiotics and future treatments

The abovementioned interactions between the HIV virus, the host gastrointestinal tract epithelium, and the microbiota have led to the development of treatments targeting microbiota-related manifestations or modifying the gut microbiota, focusing on oral or vaginal probiotic supplementation and aiming to improve disease status. Probiotic supplementation is expected to have an effect on CD4+ count, HIV infection manifestations such as diarrhea, weight loss, and cardiovascular diseases, or in the reversal of failure to thrive (a common HIV manifestation in resource-poor countries) in HIV-positive children, which remains a major concern in the ART era [66]. Trois et al. [67] assessed CD4+ count in 77 HIV1-infected children supplemented with either a *Bifidobacterium bifidum*- and *Streptococcus thermophilus*-enriched formula or standard formula over a 2-month period, and observed that, following the supplementation period, the mean CD4+ T cell count increased by 118 cells/mm^3^ to 791 cells/mm^3^ in the probiotic group, while it decreased by 42 cells/mm^3^ to 538 cells/mm^3^ in the control group. Similarly, Cunningham-Rundles et al. [66] assessed the progress of 14 children of them 12 diagnosed with failure to thrive prior to and following ART treatment for at least 1 month with subsequent *Lactobacillus plantarum* 299v (Lp299v) administration compared to controls, and demonstrated that improvement in weight was significant when comparing the pre- and post-treatment periods. However, these results may also stem from the recurrent weight loss observed in the non-responder group.

While ART treatment has increased the life expectancy of HIV-positive individuals, chronic inflammation remains central in HIV pathogenesis and morbidity. A recent study [68] followed 20 HIV-positive combined ART-treated individuals that consumed a probiotic supplement consisting of *Streptococcus*, *Bifidobacteria*, and *Lactobacillus* twice daily for 48 weeks. A variety of immune activation markers (e.g., CD4+, CD8+, CD38+, and HLA-DR+ lymphocyte counts and high-sensitivity C-reactive protein) were evaluated before and after the probiotic consumption period and were compared to those of healthy controls that did not consume any probiotics. After 48 weeks of probiotics consumption, many of the immune activation markers were significantly reduced to a level that was comparable to the marker level in the control group, including a moderate, non-significant increase in the CD4+ T cell count and a significant reduction in CD4 + CD38 + HLA-DR+ and CD8 + CD38 + HLA-DR+ T cell percentages. While IL-6 and C-reactive protein levels did not differ before and after probiotic treatment, the high-sensitivity C-reactive protein was found to significantly decrease post-treatment values compared with those prior to treatment. These findings suggest that long-term probiotics consumption may reduce some of the HIV inflammatory markers, potentially improving the chronic inflammation associated with chronic disease and ART treatment in HIV-positive individuals.

## Conclusions

Since the introduction of HAART two decades ago, the characteristics of the HIV epidemic have changed considerably, with a dramatic reduction noted in both morbidity and mortality coupled with the emergence of new chronic clinical manifestations and treatment-associated complications. While the control of HIV infection and its related morbidity remains a major challenge in many regions of the world, treatment of HIV-positive individuals in developed regions is shifting towards the long-term control of multiple co-morbidities associated with chronic HIV infection and treatment. Newly emerging chronic manifestations of HIV include a number of metabolic disorders such as cardiovascular disease, chronic hepatic and renal abnormalities, non-HIV defining cancers, osteoporosis, and growth abnormalities in young children. To date, it is well accepted that inflammation and immune dysfunction are the main inducers of these non-AIDS-associated age-related clinical manifestations [51, 62]. Nevertheless, much remains to be explored on the roles that microbiota play in HIV infectivity, persistence, and drug responsiveness. Equally interesting are the effects of microbiota in the chronic manifestations of HIV. An understanding of these roles and effects, as well as their molecular mechanisms, may offer new insights into HIV biology and potentially introduce new microbiota-associated therapeutic targets to HIV infection and its associated co-morbidities.
